# Analysis of an interprofessional education activity in the occupational health field*

**DOI:** 10.1590/1518-8345.3228.3247

**Published:** 2020-04-17

**Authors:** Ana Paula Griggio, Jaqueline Alcântara Marcelino da Silva, Rosana Aparecida Salvador Rossit, Debora Bessa Mieiro, Fernanda Maria de Miranda, Vivian Aline Mininel

**Affiliations:** 1 Universidade Federal de São Carlos, Grupo de Gestão, Formação, Saúde e Trabalho, São Carlos, SP, Brazil.; 2 Scholarship holder at the Coordenação de Aperfeiçoamento de Pessoal de Nível Superior (CAPES), Brazil.; 3 Universidade Federal de São Carlos, Departamento de Enfermagem, São Carlos, SP, Brazil.; 4 Universidade Federal de São Paulo, Centro de Desenvolvimento do Ensino Superior em Saúde, São Paulo, SP, Brazil.; 5 Universidade Federal de São Carlos, São Carlos, SP, Brazil.

**Keywords:** Occupational Health, Interprofessional Relations, Integrality in Health, Health Human Resource Training, Primary Health Care, Higher Education, Saúde do Trabalhador, Relações Interprofissionais, Integralidade em Saúde, Capacitação de Recursos Humanos em Saúde, Atenção Primária à Saúde, Educação Superior, Salud Laboral, Relaciones Interprofesionales, Integralidad en Salud, Capacitación de Recursos Humanos en Salud, Atención Primaria de Salud, Educación Superior

## Abstract

**Objective:**

to analyze the results of an Interprofessional Education activity in the ​​Occupational Health field.

**Method:**

this is an Action Research, which encompassed the implementation and evaluation stages of the activity. It was developed in a Public Higher Education Institution through 15 meetings, totaling 60 hours. It had 16 participants, five undergraduate students, three graduate students, five teachers and three health professionals, representing the areas of Nursing, Physical Education, Physiotherapy, Gerontology and Psychology. Data regarding the implementation of the activity were collected in a field diary and analyzed through Thematic Analysis. An evaluation form constructed exclusively for this purpose was applied, whose data were submitted to descriptive statistical analysis.

**Results:**

three thematic categories were identified: (1) Comprehensive care; (2) Work as a social determinant of the health-disease process; and (3) Interprofessional teamwork. The activity of Interprofessional Education was positively evaluated by the participants, who pointed out the contributions of this strategy in the construction of knowledge directed to Occupational Health.

**Conclusion:**

the activity of Interprofessional Education proved to be possible and important in the context of the formation of health professions to strengthen occupational health care.

## Abstract (pt)

**Objetivo:**

analisar os resultados de uma atividade de educação interprofissional na área de saúde do trabalhador.

**Método:**

trata-se de uma pesquisa-ação, que contemplou as etapas de implementação e avaliação da atividade. Foi desenvolvida em uma instituição pública de ensino superior em 15 encontros, totalizando 60 horas. Contou com 16 participantes, sendo cinco estudantes de graduação, três de estudantes de pós-graduação, cinco docentes e três profissionais da rede de saúde, representativos das áreas de enfermagem, educação física, fisioterapia, gerontologia e psicologia. Os dados referentes à implementação da atividade foram coletados em diário de campo e analisados por meio de análise temática. Aplicou-se um formulário avaliativo construído exclusivamente para este fim, cujos dados foram submetidos à análise estatística descritiva.

**Resultados:**

identificaram-se três categorias temáticas: (1) integralidade do cuidado; (2) trabalho como determinante social do processo saúde-doença; e (3) trabalho em equipe interprofissional. A atividade de educação interprofissional foi avaliada positivamente pelas participantes, que apontaram as contribuições desta estratégia na construção de conhecimentos direcionado à saúde do trabalhador.

**Conclusão:**

a atividade de educação interprofissional se demonstrou possível e potente no contexto da formação das profissões de saúde para fortalecimento da atenção à saúde do trabalhador.

## Abstract (es)

**Objetivo:**

analizar los resultados de una actividad de Educación Interprofesional en el área de la Salud del Trabajador.

**Método:**

se trata de una Investigación-Acción, que contempló las etapas de implementación y evaluación de la actividad; esta fue desarrollada en una Institución Pública de Enseñanza Superior en 15 encuentros, totalizando 60 horas. Contó con 16 participantes, siendo cinco estudiantes de graduación, tres estudiantes de postgraduación, cinco docentes y tres profesionales de la red de salud, representativos de las áreas de Enfermería, Educación Física, Fisioterapia, Gerontología y Psicología. Los datos referentes a la implementación de la actividad fueron recolectados en un diario de campo y analizados por medio del Análisis Temático. Fue aplicado un formulario evaluativo construido exclusivamente para este fin, cuyos datos fueron sometidos al análisis estadístico descriptivo.

**Resultados:**

fueron identificadas tres categorías temáticas: 1) la Integralidad del cuidado; 2) el Trabajo como determinante social del proceso salud-enfermedad y 3) el Trabajo en equipo interprofesional. La actividad de Educación Interprofesional fue evaluada positivamente por las participantes, las que apuntaron las contribuciones de esta estrategia en la construcción de conocimientos dirigidos a la Salud del Trabajador.

**Conclusión:**

la actividad de Educación Interprofesional se demostró posible y potente en el contexto de la formación de las profesiones de la salud, para el fortalecimiento de la atención a la Salud del Trabajador.

## Introduction

The remodeling of the training processes of health professionals has been highlighted, from the perspective of providing comprehensive care, focused on the complex individual and collective health needs^(^ 1 ^)^.

Actions have been implemented to qualify professional training through the articulation of education and professional practice in different areas. The main objective is to develop knowledge, skills, attitudes and values ​​based on collaboration and aligned with the principles of the Brazilian Public Healthcare System (SUS) and the demands of users, families and communities^(^ 2 ^)^.

Interprofessional Health Education (IPE) is a powerful strategy for articulating actions and services for the promotion, prevention, treatment and recovery of the health of individuals and communities. Its conjectures are recognized and supported by the World Health Organization (WHO) and are strengthening of the SUS principles^(^ 3 ^-^ 5 ^)^.

IPE is defined as a proposal in which members of more than one profession learn together, in order to contribute to the health and well-being of users; this interactive learning requires participation and active exchanges between members of various professions^(^ 3 ^)^, with the explicit purpose of developing contribution^(^ 5 ^)^.

Considering the potential of IPE to structure interprofessional and collaborative work and the field of Occupational Health as an interdisciplinary, multiprofessional, interinstitutional and intersectoral practice that goes beyond the limits of the health sector^(^ 6 ^)^, we sought to structure an IPE activity that would foster discussions in this theme and, thus, strengthen the training focused on the comprehensiveness of care of the working population.

Occupational Health is a constitutional right attributed to the SUS, which should be contemplated in all services of the Health Care Network (RAS), especially in Primary Care, through various policies and strategies that guide comprehensive and qualified care to worker^(^ 6 ^-^ 8 ^)^. However, these have not been shown to be sufficient to meet workers’ health needs due, among other factors, to their professional unpreparedness in dealing with specific Workers’ Health Surveillance issues, which constitute a knot in training and professional qualification^(^ 9 ^)^.

Another issue that should be considered is the transversal outline of the Occupational Health field, which requires articulated and coordinated actions between professionals and services for the effectiveness of care. However, what has been observed in practice is the fragmentation of health care, shaped by the overlapping of isolated and unrelated acts, a reflection of a uniprofessional training model, with little interface in teamwork, an aspect that hinders the establishment of common goals, as opposed to the proposal for interprofessional.

Understanding the potential of IPE in the training of students and professionals in the field of Occupational Health, this research was based on the IPE framework proposed by Barr^(^ 10 ^)^ and adopted by the WHO, which advocates the training of health professionals with more aptitude for effective teamwork and consequent achievement of collaboration in health work.

Interprofessional collaboration is defined by the sharing of responsibilities, interdependence, clarity of roles and objectives, a way of working in which identity sharing and integration is less important than in teamwork^(^ 11 ^)^.

In order to support the object of study, searches were conducted in the literature focusing on the use of IPE strategies for training in the ​​Occupational Health field and no results were found. Thus, considering the scientific and social relevance of this initiative from the perspective of interprofessional health education for the care of SUS workers and considering the possible advances from this experience, which may motivate other researchers to follow similar paths, this study was justified.

Given this contextualization, the present study aimed to analyze the results of an IPE activity developed focusing on the Occupational Health field.

## Method

This is a qualitative, descriptive and exploratory study, based on the Action Research method^(^ 12 ^)^, carried out in two sequential steps: (1) planning of the IPE activity and (2) implementation and evaluation of this activity.

Action Research is a type of social research in which the object of study is constituted by social situations and/or collective problems, allowing the active involvement of several participants through reflections, problematizations, actions and joint proposals, with the purpose of find weaknesses, propose solutions and directions aimed at overcoming or solving these situations and problems^(^ 12 ^-^ 13 ^)^.

The results presented in this manuscript are from the second study stage, which contemplated the implementation and evaluation of the IPE activity, based on previously planned planning, also based on the interprofessional principles^(^ 14 ^)^.

The activity took place in the last trimester of 2017, in a Public Higher Education Institution (IPES), in the state of Sao Paulo. It had a total workload of 60 hours, being developed in 15 weekly meetings lasting four hours each, at hours that favored the possible participants. Of these meetings, nine were face to face and six were held remotely, with activities directed through the virtual learning environment provided by the institution.

Dissemination and invitation to the activity were made through institutional mailing (email sent daily to teachers, undergraduate and graduate students and other public servants of IPES research field); on virtual social networks (Facebook and WhatsApp); and through posters placed in health departments and high circulation points in higher education institutions in the municipality (such as a university restaurant, cafeterias, bus stop) and in various points of the municipal RAS (hospitals, basic health units, family health units).

The activity’s target audience was composed by teachers, undergraduate and postgraduate students in the health area, as well as professionals who were linked to the public or private health care network. Inclusion criteria were: teaching health courses, undergraduate or postgraduate health students or public or private health professionals; to fill out the perform online application; participate in at least 75% of the proposed activities; turn in a case study at the end of the activity. Participants who did not meet the mentioned criteria were excluded. Thirty vacancies were offered, but there were only 21 enrolled, and not all met the defined inclusion criteria.

The interprofessional meetings were guided by active learning methodologies, considering the IPE precepts, such as the construction and collective problematization. In all the meetings, besides the participants, an observer and a moderator with experience in the themes and in the facilitation of groups participated. The first was responsible for recording the observed data in a field diary and the second for triggering and moderating discussions and other activities.

The activities were carried out in classrooms and laboratories provided by IPES, as well as in a virtual learning environment, in a discussion forum with previous readings and triggering questions, always facilitated by the moderator. The themes previously proposed in the planning stage contemplated: 1. Interprofessional Education; 2. Health Care Models; 3. Comprehensiveness of care; 4. National Humanization Policy; 5. Unified Health System; 6. RAS; 7. Worker’s Health Care Network; 8. Interprofessionality; 9. Occupational risks and illness processes; 10. Epidemiological profile; 11. APS (Primary Health Care) and Occupational Health Surveillance; 12. Recognition of causal link^(^ 14 ^)^.

The face-to-face and virtual meetings were recorded in a field diary, written in a word processor on a portable computer by the observer, who had the ability to type at high speed, which allowed the reproduction of information and even speech of the participants.

Data from the field diary were analyzed through Thematic Analysis^(^ 12 ^)^ and organized into three categories, which aligned with the three interprofessional competencies for occupational health care previously established in the planning stage^(^ 14 ^)^.

Thematic Analysis facilitates the understanding of the actors’ language and aims to discover the meaning of the terms spontaneously used by them and which are related to the relevant themes of the investigated situation^(^ 12 ^)^. Thus, exhaustive reading of the records in the field diary and grouping was performed according to the repetition of terms, expressions and meanings, which originated the thematic categories.

At the end of the IPE activities, participants received a five-point Likert scale virtual assessment form: strongly agree; agree; neither agree nor disagree; disagree and strongly disagree to assess: (i) the achievement of the proposed objectives; (ii) the usefulness of shared information; (iii) new knowledge in the topics addressed; (iv) learning based on the proposed objectives and (v) the structure, organization, development and methodological strategies used. The form also had four open questions, encompassing (a) what surprised you most, (b) what you appreciated most; (c) least appreciated and (d) suggestions. The data from the evaluation form were analyzed using descriptive statistics and grouping of the answers to the open questions.

The research followed the precepts of Resolution CNS 510/2016 with the approval of the Research Ethics Committee, under Opinion n. 2.291.292 and CAAE n. 68957817.5.0000.5504 and signing of the Informed Consent Form by all study participants.

## Results

The implementation and evaluation of the IPE activity had a total of 16 participants, as five participants did not meet all the inclusion criteria. All were female, being: five undergraduate students (two from Nursing, two from Gerontology and one from Psychology courses); three post-graduate students (two from the Nursing Post-graduate Program and one from the Physical Therapy Post-graduate Program), five teachers (two nurses, two physiotherapists and one physical educator) and three professionals from the municipal RAS (two hospital care nurses and a primary care nursing).

The meetings were guided by active learning methodologies such as problematization, inverted classroom, group dynamics, workshop, simulation, discussion with triggers and presentation of case study developed in groups by participants during the activity. Participation in an international event on the theme of IPE, developed at the Institution itself, was also part of the activity.

The data recorded in the field diary were analyzed and organized into thematic categories, which converged with the three competences previously established in the activity, namely: (1) Comprehensive care; (2) Work as a social determinant of the health-disease process; and (3) Interprofessional teamwork. The data from the evaluation form, applied at the end of the activity, will be presented after the results of the analytical categories.

Regarding the comprehensiveness of care, it was observed that in the first activity, which took place in a virtual environment, the participants were invited to reflect, based on the readings available and their previous knowledge, about the characteristics of the Brazilian health care model and its influence on Worker’s Health.

These discussions pointed to the persistence of the hegemony of the biomedical model, which directly influences health care, teamwork and the practice of health professionals. The emphasis on disease and biological aspects, high rates of hospitalization and unrestricted use of hard technologies and advanced resources have fragmented workers’ health care, resulting in late and timely interventions on the health of individuals, which overload the tertiary level of care.

Participants also recognized the existence of efforts to overcome this model, towards the model of comprehensive care and articulation of promotion, prevention, assistance and rehabilitation, which considers the uniqueness of the worker and health needs. This model, according to the statements, seeks to qualify care by considering the individual as a whole, taking into account the leading role of the subjects in the co-responsibility for care and the social determinants of health, with emphasis on working conditions and employability, occupational risks and illnesses related to work.

The statements highlighted many challenges found in daily work practice to consolidate the comprehensive health care model, highlighting the strong influences of the biomedical model on health work processes and interprofessional relationships. Listed, as examples, the professional unpreparedness in understanding and meeting the demands of workers beyond the disease; the knowledge of the population and the professionals themselves regarding health promotion and disease prevention; failures in the referral and counter-referral system; the lack of investments in training actions and professional training; as well as work overload and lack of physical and material resources.

Instigated by the moderator to point out strategies to overcome this reality, they indicated as potentialities: permanent and continuing education, with appreciation of the various professionals and investments in training based on interprofessional teamwork; the dissemination of information and guidance to the population about health care; understanding about the network organization of health services; the approach between professional managers and users; investments in improving the environment and working conditions.

The theme integrality of care came up several moments throughout the activity, and participants’ understanding of the coexistence of care models with divergent assumptions was notorious, which directly impact the fragmentation of professional actions and, consequently, affect the care to workers. Participants emphasized the need for investments in all spheres of SUS to change this reality and also of professionals themselves to build interprofessional collaboration that, in fact, meets the individual in its complexity.

The debates on models of attention raised questions related to the determinants and conditioning factors of the health-disease process, thus emerging the second thematic category: work as a social determinant of the health-disease process.

Reports recorded in the field diary showed that looking at the influence of work on individuals’ illness is challenging, an expression often repeated by participants. On the other hand, some worker-participants’ experiences were shared, which favored the understanding of work as an important determinant of the individuals’ health.

Many times, debates converged on the invisibility of Occupational Health in resumes and in training, which was pointed out as fragile in this regard. It was also recognized that despite the legal framework of SUS to care for workers, there are still failures in the provision of services and little recognition of the specific demands of this population by health professionals.

The suggested readings touched the participants to reflect the attention to Occupational Health in the context of Primary Care, as the entrance choice of the worker in the SUS. Thus, they pointed that teams need to be prepared to receive, host and care for workers in their particular circumstances and that the National Network of Integral Attention to Occupational Health and the National Policy for Occupational Health are great achievements for workers in this sense. However, they confirmed the need for investments in training and qualification of health professionals to consolidate such policies and programs, pointing out that initiatives such as this educational activity should be disseminated throughout the RAS.

From the perspective of the participating health professionals, the Occupational Health services are parallel to the RAS, not being linked with other points and lines of care network, and are still seen as medical specialty services.

To sensitize the participants about the relevance of work in determining the health-disease process, a simulation laboratory workshop was held with three workstations that simulated cargo loading activities; monotonous and task-focused activities; and sitting work with data entry, all designed with common goals to be achieved in a team, with time and rotation control among the participants. This experience enabled reflections on occupational exposure and illnesses resulting from work, most of the time, not perceived and reported by the worker and ignored by health professionals. It also showed that the illness can be physical and mental, especially due to the pressure for productivity, strict control and overload, complexity that requires attention and interprofessional communication during consultations, home visits and establishment of care plan.

Participants emphasized the importance of collaborative teamwork and the need for engagement of all participants to recognize the influences of work on workers’ health status and consideration of these participants in interventions. They pointed out that, in their work routine, they notice weaknesses in reception, qualified listening and effective communication between team members and between network services, which result in failures in referral and counter-referral of workers.

Overcoming the challenges found in health services for worker care requires the collective construction of coping strategies, including the user, families and communities. They considered that the themes debated in this activity should be included in vocational training as well as in-service education, ensuring the development of specific skills to meet the needs of workers.

Discussions about the challenges and potentialities of teamwork to address health in its entirety, considering the social determinants of health, resulted in the third thematic category of analysis: interprofessional teamwork.

In addition to the texts and discussions, the participants would experience two dynamics, at different times, to reflect on the importance of teamwork. Based on the different strategies, they pointed out that the comprehensiveness of care is based on a professional practice focused on the plurality of the subject, its centrality in the care and the establishment of common goals, prerequisites for teamwork based on effective communication, complementarity of actions and recognition of professional roles.

Recognition of professional roles and competencies was considered substantial for interprofessional relationships and collaboration, and teamwork goes beyond “working together”. It means looking at others with recognition of their contribution to health care, seeking communication based on respect and empathy, collective resolution of difficulties found in the work process, adding efforts to solutions that respond to user demands, considering their leading role. They confessed that they often ignore such aspects in professional practice, recognizing fragility and demonstrating readiness for change, especially motivated by team dynamics.

Participants acknowledged that IPE is a strategy that contributes to the development of competences based on the understanding and appreciation of professional roles, facilitating dialogue and effective communication, collaborative teamwork, the search for conflict resolution and co-responsibility in care, can contribute significantly to the comprehensive care to the worker as a SUS user.

In general, the three categories of analysis converged to the relevance of interprofessional collaboration in the promotion of Occupational Health, through IPE, a powerful training model that contributes to the establishment of new forms of professional interaction and partnership.

The speeches of the participants and the experiences in the meetings showed that the understanding of work as an important determinant of health is substantial for the achievement for the workers individual and collective needs. They also revealed that interprofessional teamwork and articulation of actions are prerequisites for the care model based on comprehensive care, and advances are needed to overcome the hegemonic biomedical model, which fragments actions and compromises the quality of care assistance, as well as training proposals based on the IPE.

As proposed at the beginning of this section, data on the assessment of IPE activity in the Occupational Health field are presented at this time.

At the end of the meetings, the participants were invited to perform the evaluation of the IPE activity. When asked about the knowledge acquired in each theme addressed, most participants agreed that the themes added knowledge and learning, as shown in Table 1.

**Table 1:** Acquired knowledge pointed out by participants in the activity of Interprofessional Education in Health. Sao Carlos, SP, Brazil, 2017

Item*	Strongly Agree	Agree	Neither Agree nor Disagree
Interprofessional Education Concept	62.5%	37.5%	-
Health Care Models	50%	50%	-
Comprehensiveness of care	56.25%	43.75%	-
National Humanization Policy	37.5%	62.5%	-
Unified Health System	75%	25%	-
Health Care Networks	62.5%	31.25%	6.25%
Occupational Health Care Network	62.5%	25%	12.5%
Occupational risks and illness processes	37.5%	62.5%	-
Primary Care and Occupational Health Surveillance	62.5%	31.25%	6.25%
Causal link, illness and work	25%	62.5%	12.5%
Case Study Development	56.25%	43.75%	-
Case Study Presentation	68.75%	31.25%	-

* "Disagree" and "Strongly Disagree" options did not occur, so these columns were deleted from the table

Regarding the objectives proposed in the planning stage and shared with the participants in the initial meeting, most strongly agreed that they were contemplated by the activity of IPE, as shown in Table 2.

**Table 2:** Objectives contemplated in the activity of Interprofessional Education in Health, according to participants. Sao Carlos, SP, Brazil, 2017

Objectives*	Strongly Agree	Agree
To raise awareness about the role of different professionals in Occupational Health	75%	25%
To understand the social determination of the health-disease process	87.5%	12.5%
To develop teamwork skills	87.5%	12.5%
To recognize the epidemiological and productive profile of the population assigned to the territory	75%	25%
To understand the structure of the Health Care Network and the user's referral and counter-referral system	75%	25%
To understand the actions and advances that can be incorporated into the work process in the field of Occupational Health	75%	25%

* "Neither Agree nor Disagree", "Disagree" and "Strongly Disagree" options did not occur, so these columns were deleted from the table

Table 3 presents the participants’ responses regarding the general aspects of the development of the IPE activity, pointed out as “very good” by most participants.

**Table 3:** Participants' assessments of the general aspects of the Interprofessional Health Education activity. São Carlos, SP, Brazil, 2017

Aspects*	Very good	Good
Organization	87.5%	12,5%
Relevance	93.75%	6.25%
Moderation/ Facilitation	100%	-
Debates	93.75%	6.25%
Texts	81.25%	18.75%
Round table	75%	25%
Case Study	93.75%	6.25%
Evaluation Methods	87.5%	12.5%

* "Regular", "Bad" and "Very Bad" options did not occur, so these columns were deleted from the table

The answers to the open questions of the evaluation form, which included the most and least appreciated points in the IPE activity, as well as any suggestions, were summarized in Figure 1.

**Figure 1:** Participants' considerations about the strengths and weaknesses of the Interprofessional Health Education activity and suggestions. Sao Carlos, SP, Brazil, 2017

What surprised you most about the activity?	What did you enjoy the most about the activity?	What did you least enjoy about the activity?	Sugestions
• Possibility to know different contexts and professional profiles, previously unknown.	• Opportunity and assistance for searching epidemiological databases on Occupational Health.	• As a point to be improved, I think of the late start of meetings.	• Having the case study as a closing of the Interprofessional Health Education activity and a way of construction (meeting by meeting).
• Opportunity for communication and discussion from different perspectives and points of view.	• Group activities and dynamics, which favored interprofessionality and teamwork, sharing different perspectives.	• The activity hours.	Increasing workload.
• Quality of contents, themes and readings.	• The discussions, themes, sensitivity and empathy of the moderator and the observer.	• The length of some texts suggested for reading.	• Increasing the number of classes.
• Possibility to learn from each other.	• The teaching methodologies used.		• Conduct a field work.
• Learning about the themes.	• To understand the importance of work in the illness of the individual.		• Larger number of participants.
	• Welcoming.		Offer the activity in all semesters

## Discussion

The implementation of the IPE activity based on prior planning, built collectively on the interprofessional principles, favored the development of each meeting, focusing on the development of common professional skills for the health care of workers previously defined^(^ 14 ^)^.

Using Action Research as a methodological strategy proved to be effective for the study proposal and the field diary records allowed the monitoring of the activity, as well as the perceptions and experiences of the actors involved.

In the thematic analysis, three categories that aligned with the previously defined professional competences emerged, a reflection of the activity’s own organization, planed for development in this sense.

Many times, during the activity, the participants pointed to the concern with the integration of health actions within the SUS and their interrelation with the hegemonic biomedical care model, which differs from the proposal of comprehensiveness. The care model is a logical system for the organization of RAS, influencing care and the forms of organization of health services, the work process and the use of scientific technical means^(^ 15 ^)^.

The implementation of the care model guided towards comprehensive care and expanded health needs, in line with the SUS principles, is one of the major challenges faced nowadays^(^ 15 ^)^. This happens because there is a considerable distance between the formulation of health policies and their effective consolidation in work routine^(^ 16 ^)^.

Comprehensive health care presupposes looking at the multiple determinants of the health-disease process, implying the breaking of paradigms in the way of thinking and doing health. The participants of this study pointed out that this rupture of models is only possible through investments in training, qualification and professional updating, important strategies for reality transformation.

Health education is still based on the construction of specific and isolated professional identities, with little or no integration between different areas, in a process that is far from collaboration and teamwork^(^ 17 ^)^. In an attempt to reverse this logic, the Ministry of Health, since 2003, has been investing in training strategies based on the integration and articulation of professionals’ knowledge and practices, seeking to bring students in training closer to professional practice and daily life of health services^(^ 17 ^-^ 18 ^)^.

This teaching-service articulation is also recommended by the IPE movement that seeks to bring interprofessional training closer to health work through practical experiences in the SUS. In this sense, changes have been made in the National Curriculum Guidelines of health courses over the years, in order to ensure the development of professional skills for interprofessional teamwork, necessary for meeting individual and collective health needs.

In the field of occupational health, interprofessional collaboration reaches even greater relevance, given the specificity and transversality of this area and its relationship with the social, economic and work context. Studies have emphasized the importance of this perspective in the context of health promotion and disease prevention^(^ 9 ^,^ 19 ^-^ 20 ^)^ and, in Brazil, policies and strategies have been launched aiming at strengthening Occupational Health Surveillance actions^(^ 9 ^)^.

However, for these strategies to impact on health care, it is necessary to overcome a second challenge, related to the training of health professionals^(^ 9 ^)^, which still needs to advance in understanding the influences of work on the health of individuals. The records in the field diary evidenced the participants’ concern with the gap between training and practice, an aspect that reinforces the need to strengthen the teaching-service articulation already mentioned.

In this sense, it is necessary to reformulate health education to encompass work-related issues, enabling professionals to recognize the influence of risks and injuries in the work context and to act to ensure effective practices in health care and also in the ​​Occupational Health field^(^ 20 ^-^ 21 ^)^. This enables the complementarity of knowledge and professional practices in teamwork, in order to contribute to the qualification of user care.

Given the transversality of the Occupational Health field and its nature as an interprofessional field, IPE is extremely effective for promoting inter-professional integration and collaboration among the various health professions, for the construction and development of worker care.

Studies reinforce that IPE is able to contribute to facing the challenges found in health services such as fragmentation of care and the complexity of health needs, qualifying and improving professional practice and health care^(^ 3 ^-^ 5 ^,^ 21 ^-^ 22 ^)^.

Therefore, training guided by the logic of IPE can contribute directly to the guarantee of quality and comprehensive care to the user, training professionals able to deal with the challenges and complexities in occupational health care, which was ratified by the participants of this study.

Limitations of this study are the non-representativeness of other health professions; the low adherence of the professionals of the RAS; the data collection strategy, which could have also considered video recording. It is noteworthy that the development of professional skills for Occupational Health was not the focus of this activity, given the short time it was developed.

## Conclusion

The activity of IPE with focus on Occupational Health proved to be useful and pertinent to expand knowledge in the area and to strengthen collaborative interprofessional teamwork, essential requirements for the development of common interprofessional skills. It was positively evaluated by all participants, who emphasized individual and collective learning in the topics covered and encouraged regular re-offers, encompassing other professionals and contexts.

The results showed that there are challenges to be overcome in the context of IPE and interprofessional collaboration, which permeate the health care model focused on comprehensive care, the development of actions that encompass the multiple social determinants of health and the construction of teamwork. as well as weaknesses in vocational training.

On the other hand, IPE strategies such as the one presented in this study are powerful to transform the training of health professionals and to strengthen permanent education, contributing to the construction of interprofessional collaboration.

As advances, we point out the unprecedented proposal to promote an activity based on IPE with focus on Occupational Health through Action Research, integrating undergraduate, post-graduate students, teachers and professionals of the municipal RAS.

The activity format is replicable and also allows adjustments for other health-related themes and transversal issues, requiring the incorporation of new competency assessment processes.

It is hoped that this study will motivate researchers and health professionals to apply IPE in different areas of knowledge, integrating undergraduate students, professionals and teachers, in order to strengthen the collective construction of knowledge and inter professional collaboration.

## References

[1] Pan-American Health Organization - PAHO (2017). Interprofessional Education in Health Care: Improving Human Resource
capacity for achieve universal health. Report of the meeting. Bogota,
Colombia. 7-9 December, 2016.

[2] Toassi RFC (2017). Interprofissionalidade e formação na saúde: onde estamos?.

[3] Reeves S, Fletcher S, Barr H, Birch I, Boet S, Davies N (2016). A BEME systematic review of the effects of inter professional
education: BEME Guide No.39. Med Teach.

[4] Costa MV (2016). The interprofessional education in Brazilian context: some
reflections. Rev Interface.

[5] Reeves S (2016). Ideas for the development of the interprofessional education and
practice field: an update. J Interprof Care.

[6] Minayo Gomez C, Vasconcellos LCF, Machado JMH (2018). A brief history of worker's health in Brazil's Unified Health
System: progress and challenges. Cienc Saude Colet.

[7] Lacaz FAC (2016). Workers keep on becoming ill and dying: relationships, obstacles
and challenges for the Worker's Health field. Rev Bras Saude Ocup.

[8] Amorim LA, Lacerda e Silva T, Faria HP, Machado JMH, Dias EC (2017). Workers' Surveillance at Primary Care. learning wirh Family
Health team of João Pessoa, Paraíba, Brazil. Ciênc Saúde Coletiva.

[9] Vasconcellos LCF (2018). Worker's Health Surveillance: decalogue for taking a
stand. Rev Bras Saúde Ocup.

[10] Barr H (2015). Interprofessional Education: the genesis of a global movement.

[11] Reeves S, Xyrichis A, Zwarenstein M (2018). Teamwork, collaboration, coordination, and networking: Why we
need to distinguish between different types of interprofessional
practice. J Interprof Care.

[12] Thiollent M (2011). Metodologia da pesquisa-ação.

[13] Angelim AES, Silva CML (2016). Action Research Methodology applied to interventional actions of
the Social Assistance Reference Center - SARC I in Salgueiro - PE - Cras I,
Salgueiro - PE. Id on Line Rev Psicol.

[14] Griggio AC, Mininel VA, Silva JAM (2018). Planning an interprofessional education activity for healthcare
professions. Interface.

[15] Fertonani HP, Pires DEP, Biff D, Scherer MDA (2015). The health care model: concepts and challenges for primary health
care in Brazil. Cienc Saúde Coletiva.

[16] Kalichman AO, Ayres JRCM (2016). Comprehensiveness and healthcare technologies: a narrative on
conceptual contributions to the construction of the comprehensiveness
principle in the Brazilian Unified National Health System. Cad Saúde Pública.

[17] Netto L, Silva KL, Rua MS (2016). Competency building for health promotion and change in the care
model. Rev Texto Contexto Enferm.

[18] Dias HS, Lima LD, Teixeira M (2013). The trajectory of the national policy for the reorientation of
professional training in health in the Unified Health System
(SUS). Rev Cienc Saúde Coletiva.

[19] Hofmann DA, Burke MJ, Zohar D (2017). 100 years of occupational safety research: From basic protections
and work analysis to a multilevel view of workplace safety and
risk. J Appl Psychol.

[20] Souza TS, Virgens LS (2013). Workers' health in primary health care: interfaces and
challenges. Rev Bras Saúde Ocup.

[21] Reeves S, Pelone F, Harrison R, Goldman J, Zwarenstein (2017). Interprofessional collaboration to improve professional practice
and healthcare outcomes. Cohrane Database Syst Rev.

[22] Reeves S (2016). Why we need interprofessional education to improve the delivery
of safe and effective care. Rev Interface.

